# Supplementary material for: Survival analysis of early initiation of antenatal care visits and associated factors among pregnant women in Ethiopia
Source: PLoS One. 2024 Dec 31;19(12):e0315724. doi: 10.1371/journal.pone.0315724 (PMC11687908; doi:10.1371/journal.pone.0315724)
Supplement: Attachment — Submitted filename: Response to Reviewers.docx [file pone.0315724.s001.docx]

Title: **survival analysis of early initiation of antenatal care visits and associated factors among pregnant Women in Ethiopia**

A. **Response to Editor**

**Comment:**

Please review your reference list to ensure that it is complete and correct. If you have cited papers that have been retracted, please include the rationale for doing so in the manuscript text, or remove these references and replace them with relevant current references. Any changes to the reference list should be mentioned in the rebuttal letter that accompanies your revised manuscript. If you need to cite a retracted article, indicate the article’s retracted status in the References list and also include a citation and full reference for the retraction notice.

**Response:**

Thanks for this constructive comment, which help us to revise our reference list. Hence we have assured that no retracted papers have been cited in our reference list.

B. **Response to reviewers**

**Reviewer #3**

Thanks again for spending your golden time and providing constructive comments on this manuscript.

**Comment 1:**

Researchers need to understand the distinction between the methodology section and the results section. All explanations of statistical methods should be included in the methodology section, while the results section should only present the results. I previously requested this but it was not understood.

**Response 1:**

We have incorporated this constructive comment while preparing the revised version of this manuscript.
